# Predictive role of cardiac valvular calcification in all-cause mortality of Chinese initial haemodialysis patients: a follow-up study of 4 years

**DOI:** 10.1186/s12882-023-03076-7

**Published:** 2023-02-16

**Authors:** Yun Cheng, Zhihui Lu, Xuesen Cao, Xiaoqiang Ding, Jianzhou Zou, Huimin Jin

**Affiliations:** 1 Department of Nephrology, Shanghai Pudong Hospital, Fudan University Pudong Medical Center, 2800 Gongwei Road, Pudong, Shanghai, 201399 China; 2 Division of Nephrology, Zhongshan Hospital, Fudan University, Shanghai, PR China; 3 Shanghai Medical Center of Kidney, Shanghai, PR China; 4 Shanghai Institute of Kidney and Dialysis, Shanghai, PR China; 5 Shanghai Key Laboratory of Kidney and Blood Purification, Haemodialysis Quality Control Center of Shanghai, Shanghai, PR China

**Keywords:** Haemodialysis, Cardiac valvular calcification, Outcomes

## Abstract

**Background:**

Cardiac valvular calcification (CVC) is prevalent in haemodialysis (HD) patients. Its association with mortality in Chinese incident haemodialysis (IHD) patients remains unknown.

**Methods:**

A total of 224 IHD patients who had just begun HD therapy at Zhongshan Hospital, Fudan University, were enrolled and divided into two groups according to the detection of cardiac valvular calcification (CVC) by echocardiography. The patients were followed for a median of 4 years for all-cause mortality and cardiovascular mortality.

**Results:**

During follow-up, 56 (25.0%) patients died, including 29 (51.8%) of cardiovascular disease. The adjusted HR related to all-cause mortality was 2.14 (95% CI, 1.05–4.39) for patients with cardiac valvular calcification. However, CVC was not an independent risk factor for cardiovascular mortality in patients who had just begun HD therapy.

**Conclusion:**

CVC at baseline is an independent risk factor for all-cause mortality in HD patients and makes an independent contribution to the prediction of all-cause mortality. These findings support the use of echocardiography at the beginning of HD.

## Introduction

Chronic kidney disease (CKD) affects 14.3% of people worldwide, and its. prevalence in China was 10.8% in a national survey conducted from. 2009–2010 [1, 2]. The incidence of cardiovascular events is significantly increased in patients with CKD: Almost half of CKD patients have cardiovascular disease (CVD), a proportion 4 to 5 times higher than that in the general population [3].

Various risk factors are involved in the pathophysiology of CVD. Like hypertension, diabetes, hyperlipidaemia and other traditional cardiovascular risks, CKD is an independent risk factor for CVD [4]. Cardiovascular deaths account for 40–50% of deaths in patients with end-stage renal disease (ESRD). The mortality rate for patients receiving dialysis was 193/1000 patient-years in a recent U.S. report, with 42% of deaths attributable to cardiovascular causes compared with 26% in the normal-kidney function population [5–8]. The process of vascular calcification is significantly accelerated in patients with CKD, and vascular calcification is common even in young adults with ESRD [9]. The extent and progression rate of vascular calcification in CKD patients suggest a poor prognosis [10]. Cardiac valve calcification (CVC), including aortic valve calcification (AVC) and mitral valve calcification (MVC), is a common complication observed in ESRD patients, resulting in haemodynamic dysfunction and cardiovascular events [11]. The prevalence of valve calcification is 8 to 10 times higher in haemodialysis patients than in the normal population, with 25 ~ 59% of HD patients having MVC and 28 ~ 55% having AVC [12, 13]. As shown in a meta-analysis, CVC is correlated with higher all-cause mortality risk and cardiovascular mortality in HD patients, with hazard risks of 1.73 and 2.81, respectively [14]. Due to the impact of CVC on the mortality of HD patients, Kidney Disease Improving Global Outcomes (KDIGO) guidelines have suggested the detection of cardiac valve calcification in CKD patients for risk stratification.

There are few studies on CVC in ESRD patients who are at the start of HD. Thus, our study calculated the prevalence of CVC and related independent risk factors in patients who began HD treatment in our dialysis centre. Furthermore, we conducted a prospective cohort study of the population to evaluate the predictive role of CVC in the prognosis of these incident HD patients.

## Methods

### Study population

This prospective cohort study recruited 224 patients who began HD therapy at the Blood Purification Center, Zhongshan Hospital, Fudan University, from January 1, 2010 to October 31, 2012. Exclusion criteria: < 18 years of age, rapidly progressive kidney disease, history of chronic rheumatic heart disease, chronic liver disease, cancer, kidney transplantation, and peritoneal dialysis. All patients were of Chinese origin. The clinical data included age, sex, body mass index (BMI), smoking history and comorbidities such as hypertension (HBP), diabetes (DM), and CVDs. Patients were treated three times per week (4 h per session) with standard bicarbonate dialysate (Na^+^ 138.0 mmol/L, HCO_3_^−^ 32.0 mmol/L, K^+^ 2.0 mmol/L, Ca^2+^ 1.25 mmol/L, Mg^2+^ 0.5 mmol/L) by low-flux haemodialysis using 1.4-m^2^ dialyzers with synthetic membranes (BLS514SD;Sorin Group Italia, Mirandola, Italy and Polyflux 14L; Gambro Dialysatoren GmbH, Hechigen, Germany). The blood flow was 200–300 ml/min, and the dialysate flow was 500 ml/min. The water quality conformed to the Association for the Advancement of Medical Instrumentation standard and was examined every month. During the study, dry weight was reevaluated every month to guarantee a dry weight in every patient. In our centre, all patients on haemodialysis were advised to have a high-protein diet (at least 1.2 g/kg per day with mainly animal protein).

This study was approved by the ethics committee, Zhongshan Hospital, Fudan University, and all the patients provided written informed consent.

### Anthropometric measurements, blood collection and biochemical measurements

Height and weight were measured with the patients in light clothes and barefoot. Blood was sampled on a midweek nondialysis day from 8:00 to 10:00 a.m. Red blood cells, haemoglobin, platelets, serum creatinine (SCr), albumin, blood urea nitrogen (BUN), calcium (Ca), phosphorus (P), and lipids (total cholesterol (TC), high-density lipoprotein cholesterol (HDL-C) and low-density lipoprotein cholesterol (LDL-C)) were measured by automated procedures carried out at the Department of Clinical Chemistry, Zhongshan Hospital, Fudan University using standard methods. The concentration of high-sensitivity C-reactive protein (hsCRP) was determined using an immunoturbidimetry assay. Concentrations of intact parathyroid hormone (iPTH) and N-terminal brain natriuretic peptide (NT-proBNP) were measured by electrochemiluminescence immunoassay. Serum 25 hydroxy vitamin D (25(OH)D) was measured with a radio immunoassay kit.

### Echocardiography

Two-dimensional, M-mode and Doppler echocardiography were performed using a Philips echocardiographic machine (Philips IE33; Philips, Eindhoven, The Netherlands) with a 3.5-MHz multiphase-array probe by a single experienced cardiologist within two hours after blood sampling on a midweek nondialysis day within three months after the start of HD. Cardiac valve calcification was defined as the presence of bright echoes > 1 mm in diameter on one or more cusps of the aortic valve, mitral valve or mitral annulus. Then, patients were divided into two groups according to the existence of calcified valves: patients with and without valve calcification.

### Statistical analysis

All data are expressed as means ± SDs, medians (interquartile ranges), or frequencies, as appropriate. To compare two groups of normally distributed data, the independent-samples t test was used, whereas for skewed and categorical data, the Mann‒Whitney U test or the chi-squared test was performed. The Kaplan‒Meier method was used to assess the relationship between valve calcification and all-cause mortality, and Cox proportional hazards analysis was performed to calculate relative risks. Statistical significance was defined as a two-tailed *p* value < 0.05. All analyses were performed using SPSS version 20.0 (SPSS Inc., Chicago, IL, USA).

## Results

### Baseline characteristics of the cohort

The baseline characteristics of the two groups are presented in Table 1. A total of 224 IHD patients were enrolled (148 men, 66.1%; males:females, 1.9:1), with a mean age of 57.4 ± 15.0 years. The primary underlying kidney disease was glomerular disease (90, 42.0%), followed by diabetic nephropathy (38, 17.0%). Cardiac valve calcification was observed in 74 (47.6%) patients at the beginning of the study. There were significant differences between the CVC group and the non-CVC group in the proportion of males (*P* < 0.05), age (*P* < 0.001), albumin (*P* < 0.001), BUN (*P* < 0.05), SCr (*P* < 0.05), P (*P* < 0.05) and NT-proBNP (*P* < 0.05). Patients in the CVC group were older and had lower albumin, BUN, and serum P levels as well as higher NT-proBNP (*P* < 0.05).

**Table 1:** Baseline characteristics of the CVC group and non-CVC group

Characteristic	CVC (*n* = 74)	Non-CVC (*n* = 150)	*P*
Male [n(%)]	42(56.8)	106(70.7)	< 0.05
Age(year)	68.9 ± 9.9	51.8 ± 13.9	< 0.05
Dialysis duration (m,$\overline{\mathrm{x} }$±s)*	21.9 ± 8.3	17.4 ± 7.7	< 0.05
BMI(kg/m^2^,‾x ± s)	22.93 ± 3.15	23.22 ± 3.45	0.549
CVD [n(%)]	20(27.0)	24(16.0)	0.051
DM [n(%)]	18(24.3)	37(24.7)	0.955
HBP [n(%)]	71(95.9)	147(98.0)	0.399
Smoking [n(%)]	4(5.4)	23(15.3)	< 0.05
RBC(× 10^^12^/L,‾x ± s)	3.70 ± 0.63	3.74 ± 0.54	0.573
Hb (g/L,‾x ± s)	109.7 ± 15.5	111.6 ± 15.3	0.407
Plt (× 10^^9^/ L,‾x ± s)	192.9 ± 54.1	199.1 ± 54.5	0.426
albumin(g/L,‾x ± s)	36.4 ± 3.7	38.70 ± 3.2	< 0.05
BUN (mmol/L,‾x ± s)	22.5 ± 6.0	25.3 ± 6.0	< 0.05
SCr (μmol/L,‾x ± s)	896.9 + 216.6	1089.7 ± 268.1	< 0.05
Ca (mmol/ L,‾x ± s)	2.26 ± 0.28	2.32 ± 0.25	0.105
P (mmol/L,‾x ± s)	1.88 ± 0.62	2.11 ± 0.66	< 0.05
iPTH(pg/ml,‾x ± s)	379.7 ± 319.2	365.0 ± 268.6	0.723
25(OH)D(nmol/L)	30.2(21.2 ~ 44.1)	26.3(18.8 ~ 39.1)	0.118
TC(mmol/L,‾x ± s)	4.19 ± 0.89	4.23 ± 1.00	0.880
HDL-C (mmol/L,‾x ± s)	1.05 ± 0.40	1.06 ± 0.34	0.961
LDL-C (mmol/L,‾x ± s)	2.51 ± 0.76	2.46 ± 0.87	0.827
NT-proBNP(pg/ml)	4854(2163 ~ 10,345)	3065(1568 ~ 6009)	< 0.05
hsCRP(mg/L,‾x ± s)	6.39 ± 10.10	6.50 ± 9.23	0.936
Primary renal disease [n(%)]			
CG	21(28.4%)	69(46.0%)	< 0.05
DN	12(16.2%)	26(17.3%)	0.834
HN	7(9.5%)	5(3.3%)	0.055
PKD	7(9.5%)	10(6.7%)	0.458
Other	23(31.1%)	40(26.7%)	0.131

*BMI* Body mass index, *CVD* Cardiovascular disease, *DM* Diabetes mellitus, *RBC* Red blood cells, *Hb* Haemoglobin, *Plt* Platelets, *BUN* Blood urea nitrogen, *iPTH* Intact parathyroid hormone, *25(OH)D* 25-hydroxy vitamin D, *TC* Total cholesterol, *HDL* High-density lipoprotein cholesterol, *LDL* Low-density lipoprotein cholesterol, *NT-proBNP* N-terminal brain natriuretic peptide, *CRP* High-sensitivity C-reactive protein, *CG* Chronic glomerulonephritis, *DN* Diabetic nephropathy, *HN* Hypertensive nephropathy, *PKD* Polycystic kidney disease, *Other* Lupus nephritis, gouty nephropathy, nephrotuberculosis, chronic interstitial nephritis, lipoprotein glomerulopathy and so on

### Association of Cardiac Valve Calcification with Mortality

There were a total of 56 (25.0%) deaths over a median follow-up of 47.9 months, including 29 (51.8%) deaths as a result of CVD. Causes of death are listed in Table 2.

**Table 2:** Numbers and causes of death in the CVC group and non-CVC group

	Total (*n* = 224)	CVC (*n* = 74)	Non-CVC (*n* = 150)
No. of deaths	56(25.0)	34(45.9)	22(14.7)
Cardiovascular deaths	29(12.9)	16(21.6)	13(8.7)
Cerebrovascular accident	20	10	10
Sudden death	4	4	0
Congestive heart failure	3	1	2
Myocardial infarction	1	1	0
Arrhythmia	1	0	1
Noncardiovascular deaths	27(12.1)	18(24.3)	9(6.0)
Sepsis/infection	7	5	2
Malignancy	4	4	0
Gastrointestinal bleeding	3	1	2
Other	5	4	1
Unknown	8	4	4

**Fig. 1:**
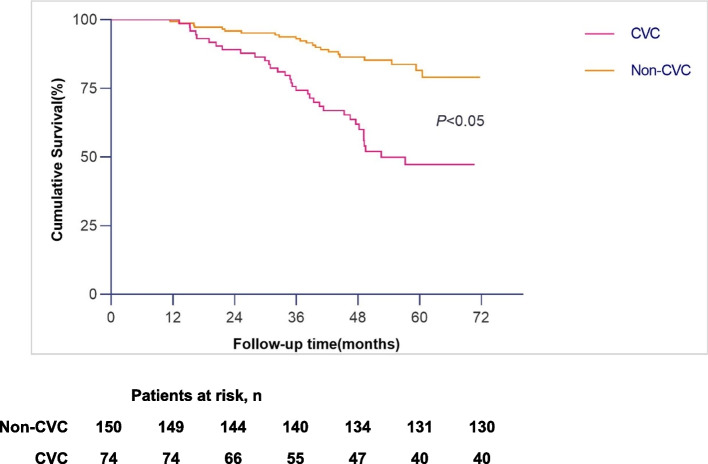
Survival curves of all-cause mortality in IHD patients in the CVC group and non-CVC group

**Fig. 2:**
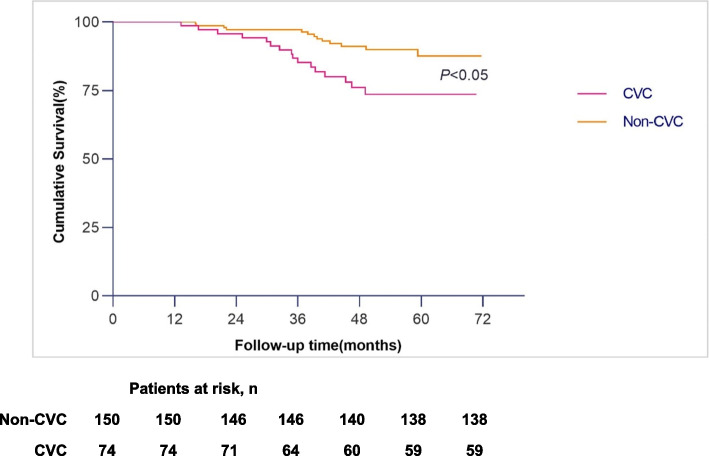
Survival curves of cardiovascular mortality in IHD patients in the CVC group and non-CVC group

Figures 1 and 2 show the Kaplan‒Meier cumulative mortality curves for patients in the CVC group and non-CVC group. All-cause mortality and cardiovascular mortality for patients with cardiac valve calcification were higher than for patients without calcification (log-rank test, *P* < 0.05 for each comparison).

Considering the traditional risk factors for death in dialysis patients and the differences in baseline data between the CVC and non-CVC groups, 16 factors, including sex, age divided by 10, BMI, history of CVD, smoking, Hb, albumin, BUN, SCr, UA, Ca, P, iPTH/10, CRP, and CVC, were adjusted by univariate proportional hazards analysis. Items with significant differences were included for Cox proportional hazards analysis. After adjustment for variables with *P* < 0.05 by univariate analysis, the prevalence of valve calcification was an independent predictor for all-cause mortality but not cardiovascular mortality (Table 3).

**Table 3:** Univariate and multivariate Cox proportional hazards analysis for all-cause and cardiovascular mortality of incident haemodialysis patients

Items	All-cause mortality		Cox proportional hazards model	
	*HR*(95% CI)	*P*	*HR*(95% CI)	*P*
Male	0.87(0.51–1.51)	0.63		
Age/10	1.67(1.34–2.09)	< 0.05	1.15(0.86–1.53)	0.34
BMI	1.01(0.93–1.10)	0.74		
History of CVD	2.11(1.19–3.75)	< 0.05	1.41(0.78–2.55)	0.26
DM	1.23(0.69–2.20)	0.48		
Smoking	0.95(0.41–2.23)	0.91		
Hb	0.96(0.95–0.98)	< 0.05	0.97(0.95–0.99)	< 0.05
albumin	0.82(0.76–0.88)	< 0.05	0.92(0.84–1.00)	0.05
BUN	0.96(0.92–1.01)	0.11		
SCr	1.00(0.99–1.00)	0.12		
UA	0.99(0.98–1.00)	0.09		
Ca	0.10(0.04–0.27)	< 0.05	0.30(0.12–0.73)	< 0.05
P	0.60(0.40–0.91)	< 0.05	1.36(0.86–2.14)	0.19
iPTH/10	0.97(0.96–0.99)	< 0.05	0.97(0.95–0.98)	< 0.05
CRP	1.00(0.97–1.02)	0.74		
CVC	3.50(2.05–5.99)	< 0.05	2.14(1.05–4.39)	< 0.05
Cardiovascular mortality				
Male	0.96(0.45–2.07)	0.92		
Age/10	1.47(1.10–1.97)	< 0.05	1.16(0.80–1.68)	0.44
BMI	1.04(0.92–1.16)	0.57		
History of CVD	2.19(0.96–4.83)	0.05		
DM	1.15(0.51–2.60)	0.74		
Smoking	1.25(0.44–3.60)	0.67		
Hb	0.98(0.95–1.00)	< 0.05	0.98(0.95–1.00)	0.08
albumin	0.89(0.80–1.00)	< 0.05	0.98(0.87–1.10)	0.68
BUN	0.98(0.92–1.05)	0.58		
SCr	1.00(1.00–1.00)	0.15		
UA	0.99(0.98–1.01)	0.24		
Ca	0.15(0.03–0.67)	< 0.05	0.35(0.10–1.26)	0.11
P	0.81(0.45–1.43)	0.46		
iPTH/10	0.98(0.96–1.00)	< 0.05	0.98(0.96–1.00)	< 0.05
CRP	0.99(0.95–1.03)	0.57		
CVC	2.79(1.34–5.80)	< 0.05	1.98(0.77–5.04)	0.15

## Discussion

CVC is regarded as an age-related degenerative disorder with little impact on heart function. Although CVC has little effect on the general population, it is an independent risk factor for all-cause mortality and cardiovascular mortality in ESRD and MHD patients [15].

Although previous studies suggest that CVC is a process of passive deposition of calcium and phosphorus on cardiac valves, growing evidence has shown that this is also an actively regulated pathophysiological process involving phenotypic transformation of vascular smooth muscle cells (VSMCs) into osteoblast-like cells [16]. Either way, CVC indicates the imbalance between promoting (advanced age, dialysis duration, diabetes, malnutrition, and minera metabolism disorder) and resisting factors (fetuin-A, pyrophosphate, and adenosine) in MHD patients [17].

In our study, patients in the CVC group had a lower percentage of males, lower albumin, lower BUN and SCr and were much older than those in the non-CVC group, which is consistent with other studies [17]. Unlike in other studies, the CVC group in our study had lower serum phosphorus. Many factors affect serum phosphorus levels, such as nutrition, dietary phosphorus intake, phosphorus binders and parathyroid function [18]. Shuvy M et al. showed that high serum levels of phosphorus are essential for CVC initiation, but after a point of no return, hyperphosphatemia is dispensable for CVC progression [19]. In our study, serum albumin and BUN levels, which represent the protein level, were significantly lower in the CVC group than in the non-CVC group. We assume that the large differences in age and albumin between the two groups affect the serum phosphorus level in the CVC group. In addition, IHD patients’ serum levels of phosphorus have already passed that point of no return, since the CKD state has been going on for a long time before dialysis begins.

After almost 4 years of follow-up, adjusting for traditional risk factors (age, sex, diabetes, smoking, albumin level, etc.), we found that CVC was an independent risk factor for all-cause mortality but not cardiovascular mortality in IHD patients, with an HR of 2.14. Our findings are consistent with previous studies about all-cause mortality, but cardiovascular mortality was not significantly higher in the CVC group, which is inconsistent with Bai’s study [15]. There may be several reasons: Bai’s study enrolled more MHD patients (434) with much longer dialysis durations than our patients (3.29 ~ 3.58 years vs. 1.45 ~ 1.83 years). Our smaller sample size and relatively shorter follow-up time may affect the result of cardiovascular mortality.

MHD patients tend to be in a state of oxidative stress and microinflammation and are prone to malnutrition and atherosclerosis, a pathology called malnutrition, inflammation and atherosclerosis/calcification (MIAC) [20]. Malnutrition (BMI and albumin) significantly affects all-cause and cardiovascular mortality in MHD patients, and malnutrition and inflammation reinforce each other [21, 22]. In our study, albumin was significantly lower in the CVC group than the non-CVC group, and multivariate Cox proportional hazards analysis also demonstrated that high serum albumin was a protective factor against all-cause (HR = 0.92, *P* = 0.05) but not cardiovascular mortality (HR = 0.98, *P* = 0.68). The mechanism by which albumin exerts its protective cardiovascular effect may be that albumin reduces the absorption of calcium, slows the apoptosis of vascular smooth muscle cells and inhibits calcification [23].

The 2012 KDIGO clinical practice guideline for anaemia in CKD recommends a haemoglobin level target of 100 to 110 g/L for HD patients [24]. Lower Hb levels (< 90 g/L) were associated with all-cause mortality. On the other hand, higher Hb levels (≥ 120 g/L) have been associated with cardiovascular mortality  [25]. When we ran multivariate Cox proportional hazards analysis, Hb level seemed to show a relatively weak protective effect on both all-cause (HR = 0.97) and cardiovascular mortality (HR = 0.98). Hb was not significantly different between the CVC and non-CVC groups in our study (109.7 g/L vs. 111.6 g/L), and the average level was approximately 110 g/L, which is the target of HD patients. Therefore, a weak protective effect might be exerted by this haemoglobin level.

Recently, some progress has been made in inhibiting CVC in HD patients. Brandenburg et al. [26] found that vitamin K supplementation can slow the progression of aortic valve calcification in HD patients. However, findings to the contrary are many; Vriese et al. [27] claimed that withdrawal of high-dose vitamin K2 in patients on haemodialysis has no significant favourable effect on VC progression. Due to the impact of CVC on the prognosis of patients with HD, we need to develop more new drugs to inhibit the progression of CVC.

There are some limitations to this study. First, it was a small, single-centre study, so no significant differences were found in some traditional risk factors for valve calcification (such as long dialysis time and hyperphosphatemia). Second, cardiac ultrasound was used to diagnose valve calcification, but it cannot accurately evaluate the severity of valve calcification; therefore, some relevant statistics could not be carried out.

## Conclusion

IHD patients have a high prevalence of CVC, and CVC is an independent risk factor for all-cause mortality in IHD patients. The shortcomings of this paper call for a large, multicentre follow-up study to clarify the impact of CVC on cardiovascular mortality in IHD patients. We suggest that regular echocardiography be performed in CKD patients, and measures should be taken to prevent CVC.

## Data Availability

The datasets supporting the current study are available from the corresponding author on reasonable request.

## Abbreviations

CVC: Cardiac valvular calcification
HD: Haemodialysis
MHD: Maintenance haemodialysis
IHD: Incident haemodialysis
CKD: Chronic kidney disease
CVD: Cardiovascular disease
ESRD: End-stage renal disease
AVC: Aortic valve calcification
MVC: Mitral valve calcification
KDIGO: Kidney Disease Improving Global Outcomes
BMI: Body mass index
BP: Blood pressure
SCr: Serum creatinine
BUN: Blood urea nitrogen
Ca: Calcium
P: Phosphorus
TC: Total cholesterol
HDL-C: High-density lipoprotein cholesterol
LDL-C: Low-density lipoprotein cholesterol
hsCRP: High-sensitivity C-reactive protein
iPTH: Intact parathyroid hormone
NT-proBNP: N-terminal brain natriuretic peptide
25(OH)D: 25 Hydroxy vitamin D
Plt: Platelets
CG: Chronic glomerulonephritis
DN: Diabetic nephropathy
HN: Hypertensive nephropathy
PKD: Polycystic kidney disease
Other: Lupus nephritis, gouty nephropathy, nephrotuberculosis, chronic interstitial nephritis, lipoprotein glomerulopathy and so on
